# Repetitive transcranial magnetic stimulation for cerebellar ataxia: a systematic review and meta-analysis

**DOI:** 10.3389/fneur.2023.1177746

**Published:** 2023-07-07

**Authors:** Lianjun Yin, Xiaoyu Wang, Lianghua Chen, Dandan Liu, Haihong Li, Zhaoxing Liu, Yong Huang, Junqi Chen

**Affiliations:** ^1^Rehabilitation Medicine, The Third Affiliated Hospital of Southern Medical University, Guangzhou, China; ^2^Department of Tuina, First Teaching Hospital of Tianjin University of Traditional Chinese Medicine, Tianjin, China; ^3^National Clinical Research Center for Chinese Medicine Acupuncture and Moxibustion, Tianjin, China; ^4^School of Traditional Chinese Medicine, Southern Medical University, Guangzhou, China

**Keywords:** cerebellar ataxia, repetitive transcranial magnetic stimulation, motor functions, meta-analysis, systematic review

## Abstract

**Background:**

Repetitive transcranial magnetic stimulation, a non-invasive brain stimulation technique, can manage cerebellar ataxia (CA) by suppressing cerebral cortical excitability. Hence, this study aimed to summarize the efficacy and safety of rTMS for CA patients by meta-analysis.

**Methods:**

The PubMed, Embase, Web of Science, and Cochrane Library databases were searched for eligible studies published till 20 May 2023. Weighted mean difference (MD) and 95% confidence intervals (CIs) were used to assess the effect of rTMS treatment. Additionally, the quality of the included studies and the risk of bias were evaluated using the Physiotherapy Evidence Database (PEDro) scale.

**Results:**

Overall, eight studies involving 278 CA patients were included in this meta-analysis. rTMS could significantly improve the Scale for the Assessment and Rating of Ataxia (SARA) (MD: −2.00; 95% CI: −3.97 to −0.02, *p* = 0.05), International Cooperative Ataxia Rating Scale (ICARS) (MD: −3.96; 95% CI: −5.51 to −2.40, *p* < 0.00001), Timed Up-and-Go test (TUG) (MD: −1.54; 95% CI: −2.24 to −0.84, *p* < 0.0001), 10-m walk test (10 MWT) (MD_10−m steps_: −2.44; 95% CI: −4.14 to −0.73, *p* = 0.005), and Berg Balance Scale (BBS) (MD: 2.59; 95% CI: 1.15–4.03, *p* = 0.0004) as compared to sham stimulation. Active rTMS was not significantly different from sham rTMS in changing the duration (MD_10−m time_: −1.29; 95% CI: −7.98 to 5.41, *p* = 0.71). No severe adverse events were observed in both sham stimulation and active rTMS groups.

**Conclusion:**

This meta-analysis provides limited evidence that rTMS may be beneficial in treating CA patients. However, these findings should be treated with caution due to the limitations of the smaller sample size and the inconsistent approach and target of rTMS treatment. Therefore, more large-scale RCTs are required to further validate our analytical findings.

**Systematic review registration:**

https://www.crd.york.ac.uk/PROSPERO/display_record.php?RecordID=295726, identifier: CRD42022295726.

## 1. Introduction

Cerebellar ataxia (CA) is a disease marked by impaired motor function that can have congenital or acquired etiologies (1). The characteristic symptoms include postural imbalance, gait disturbances, limb movement disorders, eyeball movement abnormalities, and speech impairment (2). Gait instability and poor balance contribute to the high incidence of injurious falls in CA patients (3). The prevalence of CA varies based on the etiology. Moreover, the estimated international prevalence of CA ranges between 0.3 and −3 per 100,000 (4, 5). CA may cause severe physical disabilities that impair the patient's daily living capability and burden the family and society (6, 7). The treatment of CA aims to improve the patient's motor-related abilities and quality of life. Targeted etiological treatment is the optimal treatment method for cerebellar disorders. However, the current clinical drug treatment lacks sufficient evidence of effect (8, 9). In this context, new neuromodulation therapies are urgently needed to improve the motor functions of CA patients (10).

Repetitive transcranial magnetic stimulation (rTMS) is an electrophysiological technique with neurostimulating and modulating effects (11). Due to its advantages, including high safety, non-invasiveness, and long-term neuroplasticity (12), rTMS is an alternative to the pharmacological treatment of various neuropsychiatric disorders. Through the magnetic field generated by the energized coil placed on the cranial surface, rTMS acts on cortical nerves to produce induced currents and alter the action potentials, affecting cortical excitability and promoting the neural remodeling of the targeted brain regions (13, 14). Additionally, patients are not expected to actively engage in the rTMS treatment, eliminating concerns about patient compliance and comprehension of instructions (13).

Several meta-analysis studies have demonstrated the positive effects of rTMS in the treatment of neurological diseases, including Parkinson's disease (PD) (15), Alzheimer's disease (AD) (16), epilepsy (17), migraine (18, 19), and multiple sclerosis (20). Additionally, according to several studies over the past two decades, rTMS plays an effective role in improving symptoms and facilitating recovery in CA patients (21). However, constrained by the small sample size (*n* = 1–20) of individual trials (22), it is challenging to obtain compelling evidence to affirmatively support the positive efficacy of rTMS in treating CA.

Two recent meta-analyses (23, 24) evaluated the positive effects of rTMS in CA patients. Despite these encouraging findings, certain problems were found in their study process. The previous two meta-analyses included studies published in non-peer-reviewed gray literature, such as graduation thesis, which may produce low-quality evidence. In addition, we updated the literature search to include more accumulating randomized controlled trials (RCTs). We performed a meta-analysis of published RCTs on rTMS for CA to obtain more comprehensive conclusions.

## 2. Methods

### 2.1. Search strategy

The Preferred Reporting Items for Systematic Reviews and Meta-Analyses statement was used to conduct this meta-analysis (25). The protocol was registered on the International prospective register of systematic reviews (PROSPERO) (CRD 42022295726). The PubMed, Embase, Web of Science, and Cochrane Library databases were searched for studies published in English from the inception to 20 May 2023, using the following keywords as the search terms: “Ataxia,” “Cerebellar ataxia,” “Repetitive transcranial magnetic stimulation,” and “Randomized controlled trial.” The references of included studies were also searched for potential clinical trials. Table 1 represents the search strategy for PubMed. More search strategies are available in Appendix 1.

**Table 1:** Search strategy for PubMed.

**Query**	**Search terms**
#1	“Ataxia” [MeSH Terms] OR “Cerebellar Ataxia” [MeSH Terms] OR “Spinocerebellar Ataxias” [MeSH Terms]
#2	“Ataxia” [Title/Abstract] OR “Cerebellar Ataxia” [Title/Abstract] OR “Spinocerebellar Ataxias” [Title/Abstract] OR “Cerebellar diseases” [Title/Abstract] OR “Cerebellar dysfunction” [Title/Abstract] OR “Cerebellar degeneration” [Title/Abstract] OR “Syndrome cerebellar” [Title/Abstract] OR “Cerebellum disease” [Title/Abstract] OR “Spinocerebellar diseases” [Title/Abstract] OR “Spinocerebellar degeneration” [Title/Abstract]
#3	#1 OR #2
#4	“Transcranial Magnetic Stimulation” [MeSH Terms]
#5	“Transcranial Magnetic Stimulation” [Title/Abstract] OR “Repetitive transcranial magnetic stimulation” [Title/Abstract] OR “rTMS” [Title/Abstract]
#6	#4 OR #5
#7	(“Randomized controlled trial” [Publication Type] OR “Controlled clinical trial” [Publication Type] OR “Randomized” [Title/Abstract] OR “Placebo” [Title/Abstract] OR “Clinical trials as topic” [MeSH Terms] OR “Randomly” [Title/Abstract] OR “Trial” [Title]) NOT (“Animals” [MeSH Terms] NOT “Humans” [MeSH Terms])
#8	#3 AND #6 AND #7

### 2.2. Inclusion and exclusion criteria

Inclusion criteria were formulated based on the Population, Intervention, Comparison, Outcomes and Study (PICOS) framework.

1. Participants: CA patients based on the clinical history and neurological examination, regardless of age, social status, or region.
2. Intervention: utilizes rTMS interventions (frequency, target location, intensity, and duration are not limited).
3. Control: control groups should be sham rTMS.
4. Outcome: changes in values of motor function scales post-therapy and adverse events.
5. Study: a prospective (randomized) controlled intervention study with pre- and post-testing.

Exclusion criteria are listed as follows:

1. The participants had other concurrent neurological conditions.
2. Repeated published study.
3. Study with insufficient data.
4. rTMS in combination with other interventions.
5. Trials with fewer than five treatment sessions, which would not be considered as a treatment course of brain stimulation.

### 2.3. Data extraction and quality assessment

The studies were examined by two reviewers, who individually retrieved the following information: (1) study characteristics (the first author, publication date, region, and diagnosis); (2) patient characteristics (age and gender); (3) study design (sample size, randomization, allocation, blinding, control, and intervention); (4) rTMS protocol (target position, intensity, frequency, number, and duration of sessions); (5) measures evaluating the motor function, including Scale for the Assessment and Rating of Ataxia (SARA), International Cooperative Ataxia Rating Scale (ICARS), Timed Up-and-Go test (TUG), 10-m walk test (10 MWT), and the Berg Balance Scale (BBS). This study was particularly concerned with the severity of ataxia symptoms using the SARA, which includes eight dimensions of gait, stance, sitting, speech, finger chase, nose-finger test, fast alternating hand movements, and heel-shin slide, with scores ranging between 0 and 40 (26). Higher scores indicate more severe patient dysfunction. SARA has been reported to have good reliability, validity, and responsiveness (27). Additionally, the ICARS scale was utilized to evaluate cerebellar deficits (28); the TUG test was used to assess functional mobility (29); the 10 MWT was used to measure walking ability in CA patients (30); the BBS was used to assess balance during the performance of functional activities (31); and (6) adverse effects. Moreover, two reviewers independently evaluated the risk of bias and study quality using the Physiotherapy Evidence Database (PEDro) scale (http://www.pedro.org.au/english/downloads/pedro-scale). The PEDro scale consists of 11 dichotomous items (either yes or no); the first item (eligibility criteria) is not scored; thus, the total score ranges from 0 to 10. Those scoring ≥6 are considered high-quality studies (32). Discussion with a third reviewer helped resolve the disagreement.

### 2.4. Statistical analysis

The statistical analyses were performed using the RevMan 5.4 software. Continuous variables (SARA, ICARS, TUG, 10 WMT, and BBS) were expressed by weighted mean difference (MD) together with 95% confidence intervals (CIs). Changes in mean and SDs were calculated using the formulas provided in the Cochrane Handbook (33). If the data were presented in a form other than mean and SDs, such as interquartile range and SEM, the corresponding formula was used for conversion (33–35). The statistically significant differences were set at *p* < 0.05. Study heterogeneity was assessed using the *I*^2^ values based on the Cochrane Handbook. The random-effects model was utilized if heterogeneity was found (*I*^2^ ≥ 50%). A further subgroup or sensitivity analysis was conducted to investigate possible causes of the heterogeneity. However, the fixed-effects model was used when the *I*^2^ values were <50%.

## 3. Results

### 3.1. Study selection

**Figure 1:**
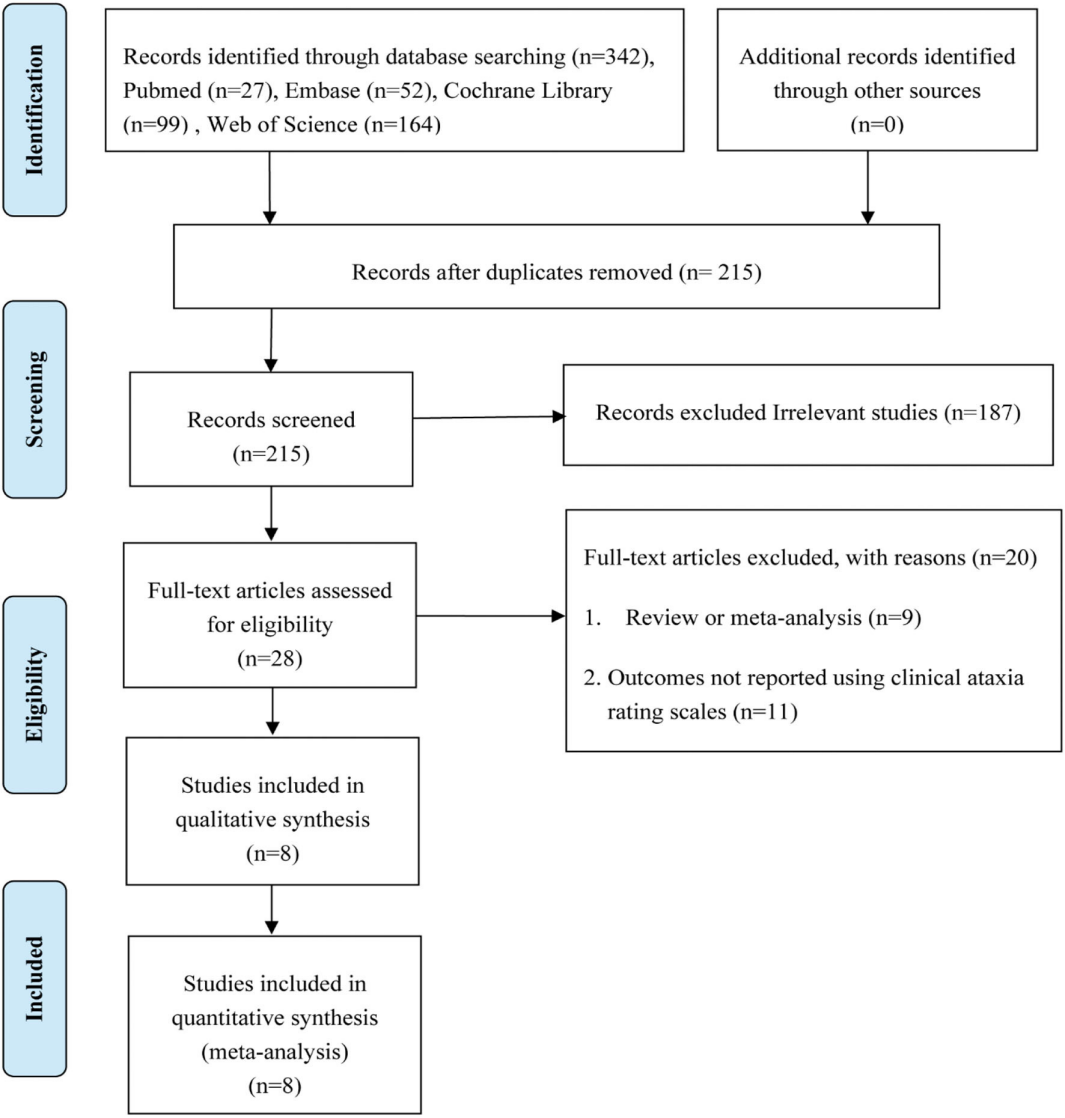
Study flow diagram.

A total of 342 studies were obtained through the initial literature search, and 127 duplicates were removed. After reading the title and abstract, 187 irrelevant studies were excluded. The remaining 28 studies were assessed by full-text reading. A total of 20 studies were further excluded as these were reviews/meta-analyses (*n* = 9) or outcomes that were not reported using clinical ataxia rating scales (*n* = 11). Finally, eight studies (36–43) were included in this meta-analysis. The flow diagram of selected studies is summarized in Figure 1.

### 3.2. Study characteristics

The included eight studies involving 278 CA patients were conducted in China (*n* = 4) (36, 41–43), Japan (40), South Korea (38), the USA (39), and Brazil (37). These studies were published between 2002 and 2023 and were conducted as double-blind and sham rTMS-controlled parallel-group designs, except for one (37) that adopted the cross-over design. Among the 278 participants, more men (51.80%) were enrolled than women (48.20%). The sample size ranged from 16 to 74, and the treatment duration varied from 5 to 25 days. Moreover, sham stimulation was administered as the control. Motor function was assessed using SARA (*n* = 5) (37, 39, 41–43), ICARS (*n* = 4) (36, 37, 42, 43), TUG (*n* = 2) (37, 39), 10 MWT (*n* = 2) (38, 40), or BBS (*n* = 2) (38, 42). The rTMS interventions in the included studies are presented in Table 2.

**Table 2:** The characteristics of the included studies in the meta-analysis.

**References**	**Country**	**Study type**	***N* (R/S)**	**Gender (M/F)**	**Population**	**Age (years) (R/S)**	**Outcomes**
Chen et al. (36)	China	Randomized, double-blind, sham rTMS controlled	9/9	8/10	SCA3	37.78 ± 9.28/ 41.78 ± 9.18	ICARS
Franca et al. (37)	Brazil	Randomized, double-blind, sham rTMS controlled, cross-over	24/24	8/16	MSA-C = 8 PCS = 7 SCA 3 = 9	53.4 ± 11.2/ 44.5 ± 15.6	SARA, ICARS, TUG
Kim et al. (38)	South Korea	Randomized, double-blind, sham rTMS controlled	22/10	17/15	PCS	64.8 ± 11.7/ 67.4 ± 7.8	10 WMT, BBS
Manor et al. (39)	United States	Randomized, double-blind, sham rTMS controlled	10/10	4/16	SCA3	53 ± 9/49 ± 4	SARA, TUG
Shiga (40)	Japan	Double-blind, sham rTMS controlled	39/35	44/30	SCA3	56.31 ± 12.24/ 58.83 ± 8.70	10 WMT
Song et al. (41)	China	Randomized, double-blind, sham rTMS controlled	25/25	29/21	MSA-c	53.1 ± 8.1/ 53.2 ± 9.4	SARA
Sikandar et al. (42)	China	Randomized, double-blind, sham rTMS controlled	22/22	24/20	SCA3	37.00 ± 9.27/ 41.84 ± 10.07	SARA, ICARS, BBS
Zhou et al. (43)	China	Randomized, double-blind, sham rTMS controlled	9/7	10/6	SCA3	39.44 ± 10.10/ 40 ± 10.18	SARA, ICARS
**References**	**Figure of coil**	**Stimulation hemisphere of cerebellum**	**Target location**	**Frequency (Hz)**	**Intensity**	**Pulse/session**	**Duration (day)**
Chen et al. (36)	F8	Bilateral	4 cm to the right of the inion, 4 cm to the left of the inion	1	100% RMT	900 pulses	One session/ day (15 total)
Franca et al. (37)	Double-cone	Contralateral	Dentate nucleus	1	90% RMT	1,200 pulses	One session/ day (5 total)
Kim et al. (38)	F8	Ipsilateral	2 cm below the inion and 2 cm lateral to the midline on the cerebellar hemisphere ipsilateral to the ataxia side	1	100% RMT	900 pulses	One session/ day (5 total)
Manor et al. (39)	C	Bilateral	4 cm lateral to the right of the inion, 4 cm lateral to the left of the inion	0.17	100% MSO	30 pulses	One session/ day (20 total)
Shiga (40)	C	Bilateral	Over the inion, 4 cm lateral to the right and left of the inion	0.17	100% MSO	30 pulses	One session/ day (21 total)
Song et al. (41)	F8	Bilateral	1 cm inferior and 3 cm left/right to the inion	50	80% RMT	1,800 pulses	One session/ day (10 total)
Sikandar et al. (42)	C	Bilateral	4 cm right of the inion, 4 cm lateral to the left of the inion	1	100% RMT	1,800 pulses	One session/ day (15 total)
Zhou et al. (43)	Double-cone	3 targets	Beginning with the right cerebellum, followed by the vermis and the left cerebellum	10	100% RMT	2,400 pulses	One session/ day (10 total)

R, real repetitive transcranial magnetic stimulation group; S, sham stimulation group; M, male; F, female; SCA3, spinocerebellar ataxia type 3; MSA-c, multiple systems atrophy cerebellar type; PCS, posterior circulation stroke; SARA, Scale for the Assessment and Rating of Ataxia; ICARS, International Cooperative Ataxia Rating Scale; TUG, Timed Up-and-Go Test; 10 WMT, 10-m time, 10-m steps; BBS, The Berg Balance Scale; F8, figure of eight; C, circular; RMT, resting motor threshold; MSO, maximum stimulator output.

### 3.3. Risk of bias

The results of the risk of bias assessment are summarized in Table 3. The PEDro scale scores for the included studies ranged from 6 to 10, with a mean score of 8.13, indicating that the included studies were of high methodological quality. Only one study (40) did not mention the application of a randomized grouping method. Moreover, only one study (37) performed concealed subject allocation.

**Table 3:** Physiotherapy Evidence Database (PEDro) scores of the studies.

**References**	**Randomized assignation**	**Concealed allocation**	**Group homogeneity**	**Participant blinding**	**Therapist blinding**	**Assessor binding**	**Dropout/15%**	**Intention-to-treat**	**Group comparisons**	**Point and variability measures**	**PEDro score (0–10)**
Chen et al. (36)	Yes	No	Yes	Yes	No	Yes	Yes	Yes	Yes	Yes	8
Franca et al. (37)^a^	Yes	Yes	Yes	Yes	Yes	Yes	Yes	Yes	Yes	Yes	10
Kim et al. (38)	Yes	No	Yes	Yes	Yes	Yes	No	Yes	Yes	Yes	8
Manor et al. (39)	Yes	No	Yes	Yes	No	Yes	Yes	Yes	Yes	Yes	8
Shiga (40)	No	No	Yes	Yes	Yes	No	No	Yes	Yes	Yes	6
Song et al. (41)	Yes	No	Yes	Yes	No	Yes	Yes	Yes	Yes	Yes	8
Sikandar et al. (42)	Yes	No	Yes	Yes	Yes	Yes	Yes	Yes	Yes	Yes	9
Zhou et al. (43)	Yes	No	Yes	Yes	Yes	No	Yes	Yes	Yes	Yes	8

Yes, scored zone point for PEDro score; No, not scored.

a Cross-over trail.

### 3.4. Meta-analysis

#### 3.4.1. Scale for the Assessment and Rating of Ataxia

**Figure 2:**
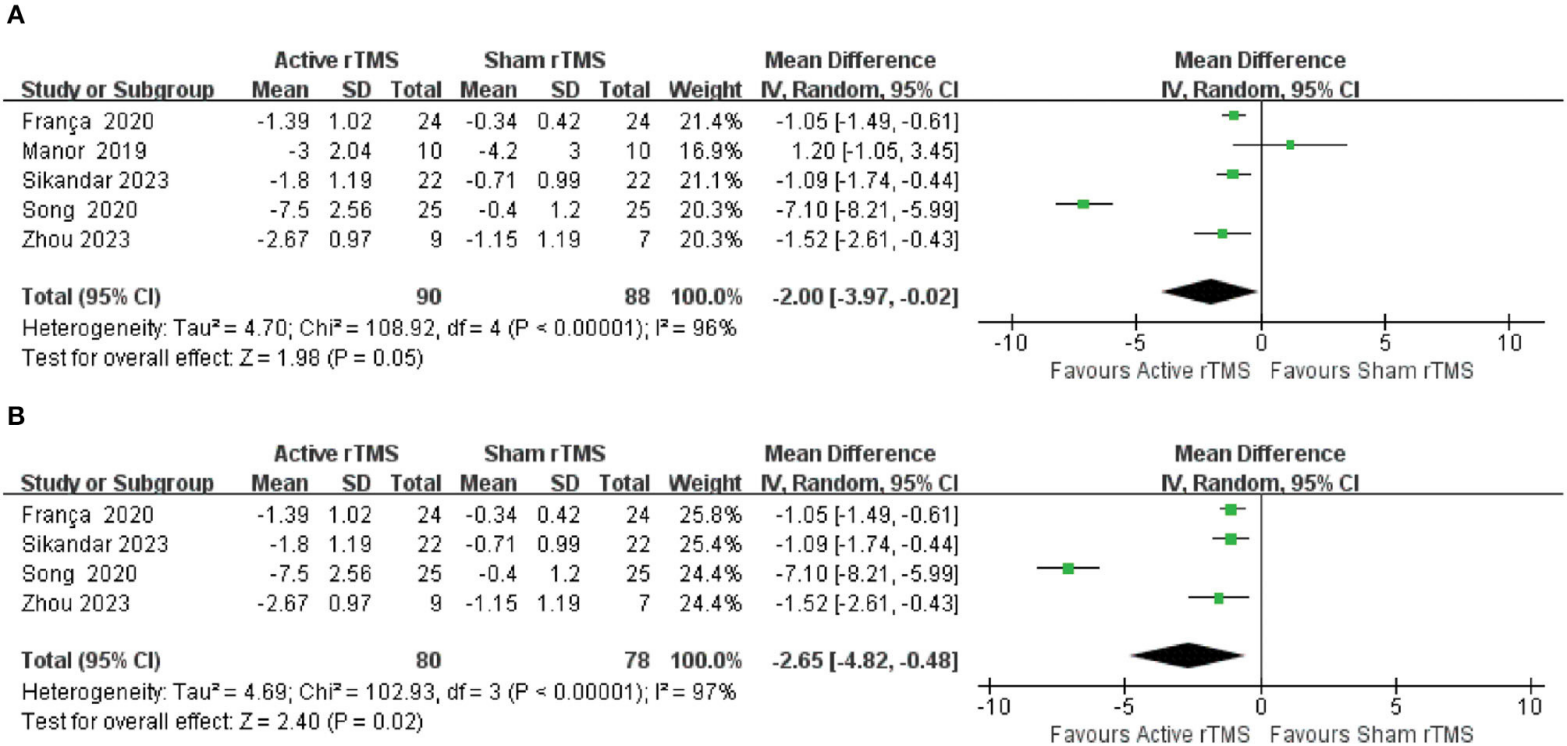
Forest plot and meta-analysis of Scale for the Assessment and Rating of Ataxia (SARA). **(A)** SARA. **(B)** Sensitivity analysis of SARA.

Five trials (37, 39, 41–43) with 154 participants compared active rTMS with sham rTMS by SARA that assessed clinical disease severity. The random-effects model was adopted because of significant heterogeneity (*I*^2^ = 96%, *p* < 0.00001). The result indicated that compared with sham rTMS, active rTMS significantly improved the SARA in CA patients (MD: −2.00; 95% CI: −3.97 to −0.02, *p* = 0. 05) (Figure 2A). In the sensitivity analysis, removing this outlier study (39) will bring the model toward more statistical significance favoring active rTMS (MD: −2.65; 95% CI: −4.82 to −0.48, *p* = 0.02) (Figure 2B).

#### 3.4.2. International Cooperative Ataxia Rating Scale

**Figure 3:**
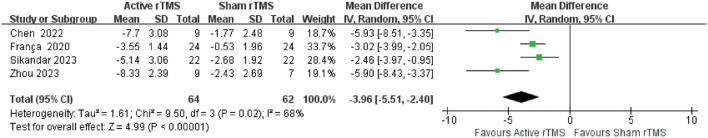
Forest plot and meta-analysis of International Cooperative Ataxia Rating Scale (ICARS).

Four trials (36, 37, 42, 43) with 102 participants compared active rTMS with sham rTMS by ICARS that assessed cerebellar dysfunction. The random-effects model was adopted because of moderate heterogeneity (*I*^2^ = 68%, *p* = 0.02). The result indicated that compared with sham rTMS, active rTMS significantly improved the ICARS in CA patients (MD: −3.96; 95% CI: −5.51 to −2.40, *p* < 0.00001) (Figure 3).

#### 3.4.3. Timed Up-and-Go test

**Figure 4:**
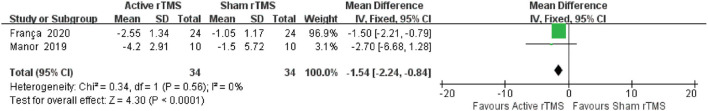
Forest plot and meta-analysis of Timed Up-and-Go test (TUG).

Two trials (37, 39) with 44 participants compared active rTMS with sham rTMS by TUG that assessed functional mobility. The fixed-effects model was adopted because of no statistically significant heterogeneity (*I*^2^ = 0%, *p* = 0.56). The result indicated that compared with sham rTMS, active rTMS significantly improved the TUG in CA patients (MD: −1.54; 95% CI: −2.24 to −0.84, *p* < 0.0001) (Figure 4).

#### 3.4.4. 10-m walk test

**Figure 5:**
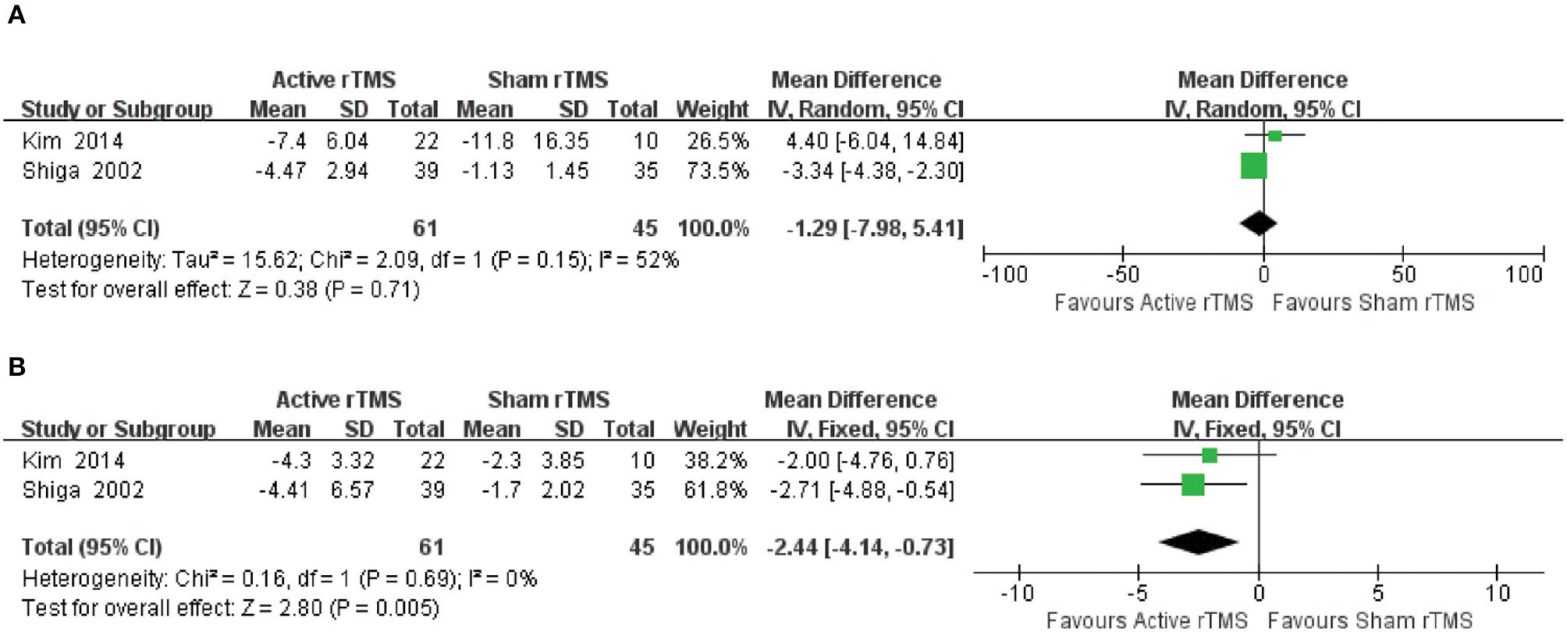
Forest plot and meta-analysis of 10 WMT. **(A)** 10-m time. **(B)** 10-m steps.

Two trials (38, 40) with 106 participants compared active rTMS with sham rTMS by 10 MWT, which measured walking ability. The result indicated that compared with sham rTMS, active rTMS showed significant changes in the CA patients in terms of the number of steps in 10 MWT (MD_10 − msteps_: −2.44; 95% CI: −4.14 to −0.73, *p* = 0.005) (Figure 5B). However, active rTMS was not significantly different from sham rTMS in changing the duration (MD_10 − mtime_: −1.29; 95% CI: −7.98 to 5.41, *p* = 0.71) (Figure 5A).

#### 3.4.5. The Berg Balance Scale

**Figure 6:**
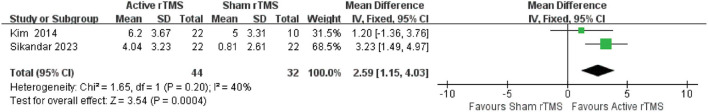
Forest plot and meta-analysis of the Berg Balance Scale (BBS).

Two trials (38, 42) with 76 participants compared active rTMS with sham rTMS by BBS that evaluated balance function. The fixed-effects model was adopted because of slight heterogeneity (*I*^2^ = 40%, *p* = 0.20). The result indicated that compared with sham rTMS, active rTMS significantly improved the BBS in CA patients (MD: 2.59; 95% CI: 1.15–4.03, *p* = 0.0004) (Figure 6).

#### 3.4.6. Subgroup analysis

**Figure 7:**
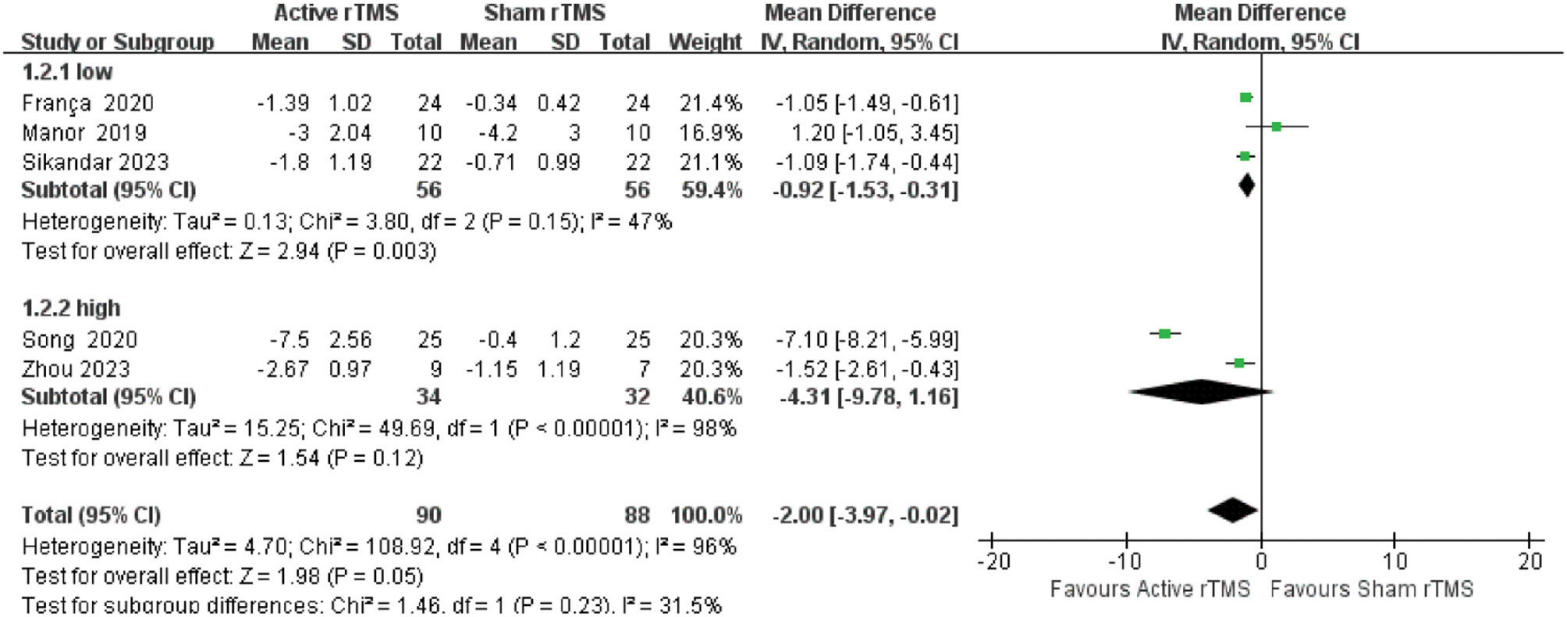
Subgroup analyses of SARA.

According to different frequencies, five studies were included for subgroup analysis. The low-frequency subgroup included (0.17 and 1 Hz), while the high-frequency subgroup included (10 and 50 Hz as iTBS). The results showed that compared with sham rTMS, low-frequency rTMS had a statistically significant improvement in SARA, with slight heterogeneity (MD: −0.92; 95% CI: −1.53 to −0.31, *p* = 0.003; *I*^2^ = 47%). In contrast, there was no difference between the high-frequency of rTMS and sham rTMS groups, with high heterogeneity (MD: −4.31, 95% CI −9.78 to 1.16, *p* = 0.12; *I*^2^ = 98%) (Figure 7).

#### 3.4.7. Safety of rTMS

**Figure 8:**
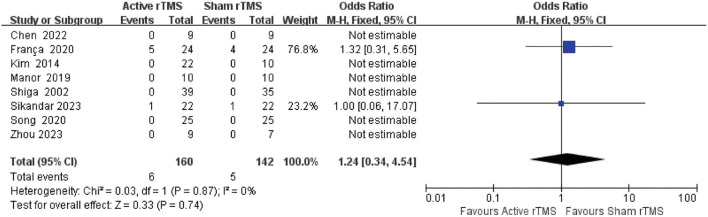
Forest plot and meta-analysis of adverse effects.

The fixed-effects model was used as there was no significant heterogeneity (*I*^2^ = 0%, *p* = 0.87) between the included studies. Two studies reported adverse events during or after treatment. There was no significant difference in the incidence of adverse events between the two groups [odds ratio (OR): 1.24; 95% CI: 0.34–4.54, *p* = 0.74] (Figure 8). Six of the included studies had no adverse events during or after rTMS treatment. A study by Franca et al. (37) reported that five patients had slight side effects during or after treatment (one felt discomfort during treatment, three had a mild headache during or after treatment, and one underwent transient worsening of the left leg pain). The study by Sikandar et al. (42) reported that nausea occurred in one patient in the rTMS group. However, all side effects resolved spontaneously after the treatment without further interventions.

## 4. Discussion

In contrast to the previous two meta-analyses (23, 24), this study included only RCTs published in peer-reviewed journals, which can yield high-quality evidence. Our study updated the literature search to include only eight articles published in English. In the previous meta-analyses, the outcome measures for motor function included SARA, ICARS, and BBS. While the current meta-analysis included further motor outcome measures, including TUG for functional mobility, 10 MWT_10 − mtime_, and 10 MWT_10 − msteps_ for walking ability. In addition, previous meta-analyses excluded special rTMS protocols, such as theta-burst stimulation (TBS) while this meta-analysis included all rTMS protocols including iTBS (41). Our findings are consistent with previous meta-analyses (23, 24) conclusions that rTMS had positively affected patients. In this study, we performed a meta-analysis of eight studies involving 278 CA patients. It proved the positive effect of active rTMS on the motor functions of CA patients. According to the study, active rTMS showed advantages in improving the clinical disease severity (SARA), cerebellar dysfunction (ICARS), functional mobility (TUG), walking ability (10 WMT_10 − msteps_), and balance function (BBS) of CA patients. However, it had no evident effect on the 10 WMT_10 − mtime_ of CA patients. Furthermore, our study proved that rTMS was safe and patients would not develop any severe adverse events other than mild pain and nausea. To summarize, rTMS has revealed inspiring potential in the clinical treatment of CA patients.

Although this meta-analysis has shown that active rTMS have beneficial effects on CA, the mechanism of rTMS has not been fully understood. The cerebellum is functionally complicated with direct or indirect relations to almost the entire central nervous system. Moreover, the dysfunction of the cerebellum and its connected neural network is considered the proximate cause of dyskinesia in CA patients (44–46). In this context, the treatments that aim to control and improve cerebellar dysfunction may have a significant clinical impact. The positive effect of rTMS in treating CA patients may be due to the action on their cerebellum, which causes lasting changes in cerebellar–thalamo–cortical pathway excitability and increases the blood flow in the cerebellar hemisphere or suppresses oxidative stress (47). The increased cerebral blood flow can activate the cerebellar functions, which have been diminished. This is proved by the investigations of Shimizu et al. (48) and Shiga (40), in which patients presented increased blood flow in the cerebellum and pontine accompanied by increased exercise volume and improved gait ataxia after rTMS treatment. In the study by Ihara et al. (47), 20 patients with spinocerebellar degeneration (SCD) received rTMS at 0.2 Hz 3 days a week for 8 weeks. The severity of oxidative stress in the central nervous system was assessed by detecting the concentration of ascorbate free radicals (AFR). According to Ihara et al., AFR levels in SCD patients decreased considerably after receiving rTMS treatment compared to healthy controls. Moreover, the decline rate was positively associated with the pretreatment AFR levels. The cerebellar facilitation effect rarely influences the motor system among patients with CA. rTMS can inhibit the excitability of the cortical motor area by acting on the cerebellar cortex and activating Purkinje cells or supplementing the insufficient inhibitory effect of the cerebellar nucleus due to the impaired or absent functions of Purkinje cells. This results in the transient facilitation of inhibitory neurons (49). Similarly, symptoms, such as gait, can be improved and motor functions can be regulated. rTMS can generate long-lasting effects in treating various complicated neurological diseases such as Parkinson's (50), Alzheimer's (51), and ataxia (52). Therefore, more research is required to determine the precise mechanism of action of rTMS for CA.

However, there are certain limitations in the current meta-analysis. First, due to strict inclusion criteria, only eight studies published in English in peer-reviewed journals were included, which inevitably led to the issue of publication bias. Second, heterogeneity is inevitable due to different stimulus locations, intervention duration, stimulus intensity, and pulse number, and this inconsistency may affect the results' validity and the study's reproducibility. Therefore, there is a need for a stimulation protocol based on evidence-based rTMS. Third, given the lack of subgroup analysis based on the ataxia subtype, gender, and age, the efficacy of rTMS should be drawn with careful deliberation. Fourth, multiple outcome measures evaluated the motor functions in CA patients in the included studies; however, only a few studies were selected for pooled analyses. Furthermore, studies have confirmed that the cerebellum plays a critical role in many cognitive and affective functions, resulting in cognitive and social deficits among CA that may significantly impact their quality of life, which were not mentioned in any of the included studies. Developing a core outcome set (COS) (53) in clinical trials concerning CA is necessary. Finally, it is challenging to assess the long-term effects of rTMS in CA patients due to limited studies that provided follow-up results.

## 5. Conclusion

The meta-analysis preliminarily indicates that rTMS has a positive effect on alleviating the symptoms of CA patients. However, these findings should be treated with caution due to the limited number of research articles, the small number of case studies in the included articles, the short duration of treatment, and the inconsistent approach and target of rTMS treatment. Further large-scale studies are needed to explore the optimal stimulus parameters.

## Data availability statement

The original contributions presented in the study are included in the article/Supplementary material, further inquiries can be directed to the corresponding author.

## Author contributions

LY, XW, and JC: conceptualization. LY, XW, and DL: methodology, software, and writing—original draft preparation. HL and ZL: study selection and data extraction. LY and YH: quality assessment. All authors have read and agreed to the published version of the manuscript.
