# Supplementary material for: Repetitive transcranial magnetic stimulation for cerebellar ataxia: a systematic review and meta-analysis
Source: Front Neurol. 2023 Jul 7;14:1177746. doi: 10.3389/fneur.2023.1177746 (PMC10360185; doi:10.3389/fneur.2023.1177746)
Supplement: Supplementary file 1 [file Data_Sheet_1.pdf]

Repetitive transcranial magnetic stimulation for cerebellar ataxia: a systematic review and meta-analysis

Supplementary Material: Search strategy

PubMed

#1	"Ataxia"[Mesh] OR "Cerebellar Ataxia"[Mesh] OR "Spinocerebellar Ataxias"[Mesh]
#2	"Ataxia"[Title/Abstract] OR "Cerebellar Ataxia"[Title/Abstract] OR "Spinocerebellar Ataxias"[Title/Abstract] OR "cerebellar diseases"[Title/Abstract] OR "cerebellar dysfunction"[Title/Abstract] OR "cerebellar degeneration"[Title/Abstract] OR "syndrome cerebellar"[Title/Abstract] OR "cerebellum disease"[Title/Abstract] OR "spinocerebellar diseases"[Title/Abstract] OR "spinocerebellar degeneration"[Title/Abstract]
#3	#1 OR #2
#4	"Transcranial Magnetic Stimulation"[MeSH Terms]
#5	"Transcranial Magnetic Stimulation"[Title/Abstract] OR "Repetitive transcranial magnetic stimulation"[Title/Abstract] OR "Noninvasive brain stimulation"[Title/Abstract] OR "TMS"[Title/Abstract] OR "rTMS"[Title/Abstract]
#6	#4 OR #5
#7	("randomized controlled trial"[Publication Type] OR "controlled clinical trial"[Publication Type] OR "randomized"[Title/Abstract] OR "placebo"[Title/Abstract] OR "clinical trials as topic"[MeSH Terms] OR "randomly"[Title/Abstract] OR "trial"[Title]) NOT ("animals"[MeSH Terms] NOT "humans"[MeSH Terms])
#8	#3 AND #6 AND #7

EMbase

#1	'Ataxia':ab,ti OR 'Cerebellar Ataxia':ab,ti OR 'Spinocerebellar Ataxias':ab,ti OR 'cerebellar diseases':ab,ti OR 'cerebellar dysfunction':ab,ti OR 'cerebellar degeneration':ab,ti OR 'syndrome cerebellar':ab,ti OR 'cerebellum disease':ab,ti OR 'spinocerebellar diseases':ab,ti OR 'spinocerebellar degeneration':ab,ti
#2	'Transcranial Magnetic Stimulation':ab,ti OR 'Repetitive transcranial magnetic stimulation':ab,ti OR 'Noninvasive brain stimulation':ab,ti OR 'TMS':ab,ti OR 'rTMS':ab,ti
#3	('randomized controlled trial':ab,ti OR ('controlled clinical trial' OR 'randomized':ab,ti OR 'randomly':ab,ti OR 'trial':ab,ti OR 'placebo':ab,ti OR 'clinical article':ab,ti OR 'clinical trial':ab,ti OR 'controlled study':ab,ti OR 'major clinical study':ab,ti OR 'double blind procedure':ab,ti OR 'multicenter study':ab,ti OR 'single blind procedure':ab,ti OR 'crossover procedure':ab,ti))
#4	#1 AND #2 AND #3

Cochrane

#1	(Ataxia):ti,ab,kw OR (Cerebellar Ataxia):ti,ab,kw OR (Spinocerebellar Ataxias):ti,ab,kw OR (cerebellar diseases):ti,ab,kw OR (cerebellar dysfunction):ti,ab,kw OR (cerebellar degeneration):ti,ab,kw OR (syndrome cerebellar):ti,ab,kw OR (cerebellum disease):ti,ab,kw OR (spinocerebellar diseases):ti,ab,kw OR (spinocerebellar degeneration):ti,ab,kw
#2	(Transcranial Magnetic Stimulation):ti,ab,kw OR (Repetitive transcranial magnetic stimulation):ti,ab,kw OR (Noninvasive brain stimulation):ti,ab,kw OR (TMS):ti,ab,kw OR (rTMS):ti,ab,kw
#3	(randomized controlled study):ti,ab,kw OR (randomized controlled trial):ti,ab,kw OR (randomized trial):ti,ab,kw OR (randomized study):ti,ab,kw OR (randomized placebo-controlled study):ti,ab,kw OR (randomized parallel-group study):ti,ab,kw OR (controlled clinical trial):ti,ab,kw OR (multicenter study):ti,ab,kw OR (double-blinded controlled study):ti,ab,kw
#4	#1 AND #2 AND #3

Web of science

#1	TS = (Ataxia OR Cerebellar Ataxia OR Spinocerebellar Ataxias OR cerebellar diseases OR cerebellar dysfunction OR cerebellar degeneration OR syndrome cerebellar OR cerebellum disease OR spinocerebellar diseases OR spinocerebellar degeneration)
#2	TS = (Transcranial Magnetic Stimulation OR Repetitive transcranial magnetic stimulation OR Noninvasive brain stimulation OR TMS OR rTMS)
#3	TS = (randomized OR randomly OR placebo OR trial)
#4	#1 AND #2 AND #3
